# *Fusobacterium nucleatum* Pleural Empyema in a Patient with Progressive Rheumatoid Arthritis and Immunosuppression

**DOI:** 10.1155/2021/5212401

**Published:** 2021-07-10

**Authors:** Wesley Tang, Zi Yu Liu, Charles Abreu

**Affiliations:** Department of Medicine, Kettering Medical Center, Dayton, OH, USA

## Abstract

*Fusobacterium nucleatum* is an anaerobic oral commensal organism that is often associated with inflammatory bowel disease, adverse pregnancy outcomes, respiratory tract infections, and Lemierre's syndrome. Rheumatoid arthritis is often associated with pleuropulmonary manifestations including noninfectious pleural effusions and interstitial lung disease. We present a case of a 47-year-old man with progressive rheumatoid arthritis on immunosuppressive therapy who was found to have a left-sided pleural effusion, thought secondary to possible pneumonia, and was treated with levofloxacin and methylprednisolone. He presented a month later and was found to have a large left-sided thick-walled fluid collection found to be an empyema. A chest tube was placed, and fluid culture grew *Fusobacterium nucleatum*. The patient was successfully treated with intrapleural fibrinolytic therapy and amoxicillin-clavulanic acid.

## 1. Introduction

Anaerobic pulmonary and pleural infections are typically associated with aspiration of saliva and upper airway secretions of gastric content. As anaerobes can be difficult to isolate and culture, they have been historically underrepresented in the literature with regard to pleural infections [1, 2]. The most pathogenic organisms described are *Fusobacterium nucleatum*, *Bacteroides melaninogenicus*, and anaerobic Gram-positive cocci [3]. *Fusobacterium nucleatum* is an anaerobic oral commensal organism that is often associated with inflammatory bowel disease, adverse pregnancy outcomes, respiratory tract infections, and Lemierre's syndrome. As an opportunistic pathogen, it is seldom isolated in immunocompetent patients. With regard to rheumatoid arthritis, pleural involvement in rheumatoid arthritis (RA) is common, and if a pleural effusion is present, it is usually exudative in quality. Consequently, it is important to determine if the exudative effusion in a patient living with RA is due to inflammation and sequalae from RA or whether the exudative pleural effusion is infected as in empyema. We present a case in which a patient with RA, with recent immunosuppression of prednisone, methotrexate, and adalimumab, presented with an exudative pleural effusion. This pleural effusion was later identified as secondary to a *Fusobacterium nucleatum* infection and empyema. There have been few case reports in the literature describing *Fusobacterium nucleatum* empyema due to the rarity of the organism and the nature of anaerobes being notoriously difficult to culture from pleural infections.

## 2. Case Description

A 47-year-old Caucasian male presented to the emergency department with complaints of a 4-day history of left-sided lateral chest wall and left shoulder pain with associated shortness of breath. His past medical history was notable for severe rheumatoid arthritis, psoriasis, coronary spasm, hypertension, and hyperlipidemia. Prior surgical history includes left ankle fracture repair, right knee arthroscopy x2, and right wrist surgery. He is a heavy smoker, up to 3 packs per day, and has been substituting cigarette smoking with vaping in recent months. The patient reported underlying severe rheumatoid arthritis with multiple joint involvement including the wrist, fingers, shoulders, and knees. He had been on prednisone as well as adalimumab before. He was recently placed on methotrexate due to progressive rheumatoid arthritis by his rheumatologist. A month prior to presentation, he underwent a right knee medial meniscectomy for a meniscus tear, and his adalimumab and methotrexate were temporarily held at that time. Lab work on presentation was significant for a C-reactive protein (CRP) of 73.6 mg/L and erythrocyte sedimentation rate (ESR) of 22 mm/hr; all other relevant lab values were within the reference range and unremarkable. Computed tomography (CT) of the chest revealed diffuse ground-glass opacities and a moderate left-sided effusion with consolidation in the left lung base. Ultrasound prior to thoracentesis confirmed the pleural fluid collection, and the patient underwent thoracentesis; 600 ml of straw-colored pleural fluid was removed, and the fluid collection was drained to dryness. Fluid analysis was suggestive of an exudative process (pH 7.11, LDH 657 U/L, protein 5.2 g/dL, glucose 11.0 mg/dL, triglyceride 48 mg/dL, and amylase 22 U/L), cytology was negative for malignancy, and fluid culture was negative. He was treated with IV levofloxacin and methylprednisolone for empiric pneumonia and possible rheumatoid-related pleuritis. He was then discharged home with levofloxacin and home-dose prednisone.

After his discharge, the patient continued to experience some shortness of breath and had persistent mild left-sided pleuritic chest pain. He additionally complained of cough with clear secretions, but without fever or chills. A month later, he again presented to the emergency department with complaints of worsening shortness of breath, fever, chest tightness, cough with clear sputum, and fatigue. During this period, he continued to take adalimumab and prednisone but had stopped taking methotrexate. Initial vital signs were remarkable for fever of 100.7 F, with stable blood pressure, heart rate, respiratory rate, and oxygen saturating on room air.

**Figure 1:**
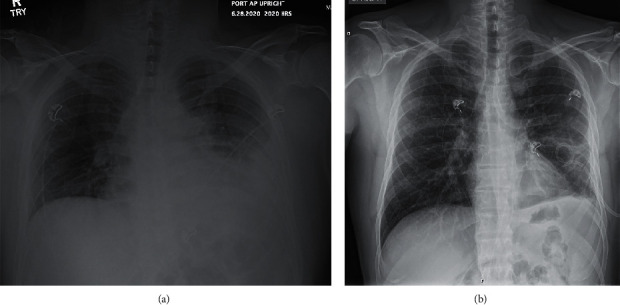
Xray before and after pigtail chest tube placement on second hospitalization.

**Figure 2:**
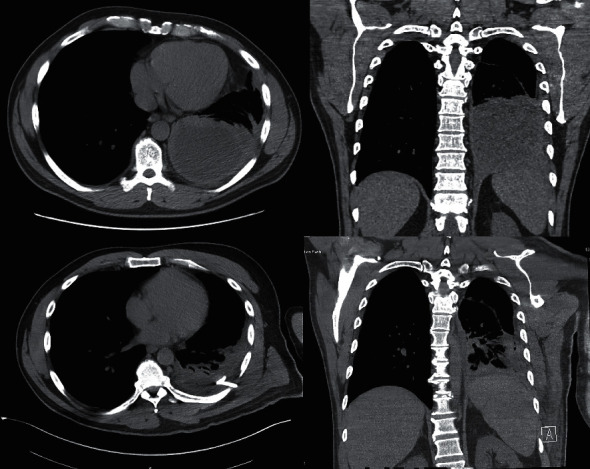
Chest CT showing left-sided pleural effusion, with follow-up chest CT demonstrating decreased fluid collection after systemic antibiotics and intrapleural fibrinolytic therapy.

Physical examination revealed decreased breath sounds in the left lower lung base. A prominent bony enlargement and mild synovitis of the distal interphalangeal joint (DIP) and proximal interphalangeal joint (PIP) with diffuse tenderness to palpation of all finger joints were noted, along with a limited range of motion of bilateral glenohumeral joints. The remaining physical examination was within normal limits. Laboratory evaluation showed leukocytosis of 13.3 k/uL, unremarkable chemistry panel, elevated CRP of 64.83 mg/L, erythrocyte sedimentation rate 78 mm/hr, and procalcitonin 0.27 ng/ml. Chest X-ray demonstrated left basilar pleural-parenchymal disease with probable loculated pleural effusion (Figure 1). CT of the chest this time showed a thick-walled fluid collection in the left lung base that, per the read, appeared intraparenchymal (Figure 2). Subsequent ultrasound showed a moderate left pleural effusion estimated at 640 mL. The patient underwent thoracentesis with chest tube placement, and 400 ml of malodorous purulent yellow/green fluid was removed. The pleural fluid was too thick to be analyzed in the lab and was sent for culture and pathology only. He was started on broad-spectrum antibiotics: vancomycin and piperacillin/tazobactam; vancomycin was discontinued after 4 days once cultures had resulted, and piperacillin/tazobactam was continued for 9 days while inpatient. Pulmonology was consulted and without the assistance of general surgery was treated with intrapleural fibrinolytic therapy with 10 mg of tissue plasminogen activator (tPA) and 5 mg of Dornase alfa for a total of 6 courses, 12 hours apart. Pathology again was negative for malignancy, and culture 4 days later revealed *Fusobacterium nucleatum*. Blood cultures were negative. Repeat chest CT (Figure 2) showed a decrease in size of the left-sided pleural effusion with a pigtail chest tube placed in the posterior left lung base. The patient was eventually discharged in an improved condition, and antibiotics were deescalated at the time of discharge to oral amoxicillin-clavulanic acid for a 14 day course, for a total of 23 days of antimicrobial therapy. At his one month follow-up with pulmonology, the patient noted symptomatic resolution and did not obtain the follow-up imaging that was ordered. The patient resumed taking adalimumab and methotrexate for rheumatoid arthritis after his respiratory symptoms resolved. At nine months after initial presentation, the patient remains symptom free.

## 3. Discussion

Rheumatoid arthritis is a chronic inflammatory disease affecting the joints, which frequently includes extra-articular effects. Pulmonary manifestations can include pulmonary nodules, interstitial lung disease, and exudative effusions [4]. The hazard ratio in developing a pleural empyema in a patient with RA may be as high as 11x compared to patients without RA [5]. The presence of frank empyema in RA is not common and is usually attributed to the breakdown of subpleural rheumatoid nodules with or without secondary infection [6]. It is important to determine if empyema in a patient with RA is due to pleural space infection or inflammatory rheumatoid flare as the treatment of the two conditions is significantly different.

Empyema, a collection of pus in the pleural cavity, is associated with a high rate of mortality. When diagnosed in the early clinical stage, it is imperative to provide antibiotic coverage for both aerobes and anaerobes. It is important to note that the incidence of anaerobic empyema has been on the rise due to more modern culturing techniques. With DNA amplification techniques, the incidence of anaerobes can be as high as 70% when compared to 20% with conventional techniques [7]. *Fusobacterium nucleatum* is an anaerobic oral commensal and a periodontal pathogen associated with a wide spectrum of human diseases [8]. Fusobacterium bacteremia is uncommon, accounting for approximately 0.7–0.9% of patients with bacteremia [9]. *Fusobacterium nucleatum* has been associated with as high a 30-day mortality rate of up to 47.4% [10]. More recently, it is increasingly associated with colorectal cancer tissues in patients with recurrence after chemotherapy [11]. With regard to pulmonary infections, *Fusobacterium* species are not normally considered to be involved in pleural empyema. There are limited numbers of case reports in the literature describing *Fusobacterium* empyema partly due to the rarity of the organism spreading to the pleural space and partly due to the nature of anaerobes being difficult to culture [12]. When isolated, organisms are found in patients with a history of preceding chest trauma, surgery, esophageal perforation, thoracentesis, pneumothorax, bacteremia, and tobacco or alcohol use [13]. In our case, when the patient first presented, he was provided levofloxacin, an antibiotic with limited anaerobic coverage. Due to inadequate coverage, it is likely the patient's disease progressed. Upon second presentation, antibiotic coverage was broadened with piperacillin/tazobactam, a chest tube was placed, and fibrinolytics were added. The addition of evacuation and drainage of the pleural space with added intrapleural therapy was likely the key to successfully treating an anaerobic pleural infection in an immunosuppressed patient. The patient would fully recover and continues to follow-up with pulmonology outpatient. We postulate that our patient's risk factors for *Fusobacterium* empyema were severe rheumatoid arthritis and immunosuppression with methotrexate, prednisone, and adalimumab, in addition to the patient's smoking.
